# Potential of the microalgae *Chlorella fusca* (Trebouxiophyceae, Chlorophyta) for biomass production and urban wastewater phycoremediation

**DOI:** 10.1186/s13568-022-01384-z

**Published:** 2022-04-15

**Authors:** María Ángeles Arrojo, Luciana Regaldo, Jesús Calvo Orquín, Félix L. Figueroa, Roberto Teófilo Abdala Díaz

**Affiliations:** 1 Departamento de Ecología. Facultad de Ciencias, Instituto Andaluz de Biotecnología Y Desarrollo Azul (IBYDA), Universidad de Málaga. Campus Universitario de Teatinos S/N, 29071 Málaga, Spain; 2 Laboratorio de Ecotoxicología. Facultad de Humanidades Y Ciencias, Universidad Nacional del Litoral. Consejo Nacional de Investigaciones Científicas Y Técnicas (CONICET), 3000 Santa Fe, CP Argentina

**Keywords:** Microalgae, *Chlorella fusca*, Nutrient removal efficiency, Urban wastewater bioremediation, Lipid, Protein

## Abstract

**Abstract:**

The present work focuses on: (1) the evaluation of the potential of *Chlorella fusca* to grow and synthesize metabolites of biotechnological interest, after being exposed for fourteen days to urban wastewater (UW) from Malaga city (UW concentrations: 25%, 50%, 75%, and 100%); (2) the study of the capacity of *C. fusca* to bioremediate UW in photobioreactors at laboratory scale; and (3) the evaluation of the effect of UW on the physiological status of *C. fusca*, as photosynthetic capacity by using in vivo Chl *a* fluorescence related to photosystem II and the production of photosynthetic pigments. *C. fusca* cell density increased in treatments with 50% UW concentration, followed by the treatment with 100% UW, 75% UW, the control, and finally 25% UW. Protein content increased to 50.5% in 75% UW concentration. Stress induced to microalgal cultures favored the increase of lipid production, reaching a maximum of 16.7% in 100% UW concentration. The biological oxygen demand (BOD_5_) analysis indicated a 75% decrease in 100% UW concentration. Dissolved organic carbon (DOC) levels decreased by 41% and 40% in 50% UW and 100% UW concentration, and total nitrogen (TN) decreased by 55% in 50% UW concentration. The physiological status showed the stressful effect caused by the presence of UW on photosynthetic activity, with increasing impact as UW concentration grew. In the framework of circular economy, we seek to deepen this study to use the biomass of *C. fusca* to obtain metabolites of interest for biofuel production and other biotechnological areas.

**Graphical Abstract:**

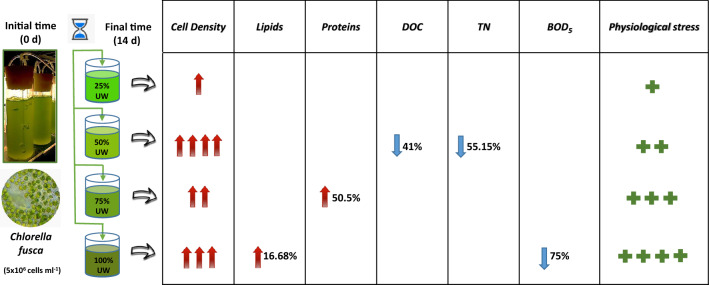

## Keypoints

- *Chlorella fusca* grows and synthesizes metabolites of biotechnological interest in UW
- Protein and lipid content increased to 50.5% and 16.7% in 75% UW and 100% UW concentrations
- TN, DOC, and BOD_5_ reduction efficiency increased with the exposure time.

## Introduction

At the international level, different countries have advanced the Sustainable Development Goals (SDG) adopted by the General Assembly of the United Nations in 2015, where two of the objectives agreed by the group were: *“Ensure availability and sustainable management of water and sanitation for all”* (SDG 6) and *“Ensure access to affordable, reliable, sustainable and modern energy for all”* (SDG 7).

These environmental policies reflect the need for advancing jointly in the optimization of effluent treatment processes in urban agglomerates and in the use of different sources of renewable energy to limit the use of fossil fuels, which generate an increase in greenhouse gases (GHGs) (Mikhaylov et al. 2020).

The urban effluents are commonly referred to as "urban wastewater" (UW) and include domestic wastewater or its mixture with industrial wastewater and/or stormwater runoff. Domestic wastewater contains effluents from housing and service areas generated mainly by human metabolism and domestic activities (European Commission 1991). They contain a very diverse physicochemical composition, from simple organic and inorganic compounds to substances that are known as "emerging contaminants", including drugs (Salibián 2014), microplastics (Woodward et al. 2021), and nanoparticles (Kühr et al. 2018). Currently, the problem is becoming more complex with the COVID-19 pandemic. Some researchers detected SARS-CoV-2 in UWs from different countries in America, Europe, Asia, and Oceania (Haramoto et al. 2020; Randazzo et al. 2020; Giraud Billoud et al. 2021, among others).

The UW treatment aims to avoid damage to public, private, and industrial water supplies, to water intended for recreation, fishing activities, agriculture, and also depreciation of the value of the land and impacts on the ecosystems and human health (Cossio et al. 2021).

In recent years, the need to contribute to the development of efficient and economically convenient methods for UW treatment has motivated the study of new technologies. In this sense, microalgal applications have increased in the last decade, due to their importance in phycoremediation and to their extensive application potential in biopharmaceutical, nutraceutical, and renewable energy industries (Khan et al. 2018; Rao et al. 2019).

Microalgae could generate a wide variety of bioproducts, including pigments, proteins, polysaccharides, lipids, vitamins, antioxidants, and bioactive compounds (Tang et al. 2020). Several species of microalgae have been investigated for their ability to synthesize and store bioproducts with pharmacological and biological properties, as well as metabolites used for biofuel production (Khan et al. 2018). Different species of the genus *Chlorella* have been described as possible sources for biodiesel production according to their oil properties (Sathish, 2020; Katiyar et al. 2021).

There are records of the use of different fractions of industrial, domestic, or urban effluents as culture media for chlorophyte microalgae (Singh et al. 2017). This is based on the presence of compounds that can be sources of organic carbon, nitrogen, phosphorus, and trace elements. Many of these compounds can be efficiently metabolized (Pacheco et al. 2015).

Compared to other biofuel feedstocks, microalgae have the following benefits: (1) They do not compete with cropland and freshwater crops because they can be grown in saline water and nonarable land (Cai et al. 2013). (2) They can grow at an exceptionally fast rate (Tredici 2010). (3) They show a high oil content of 20%-50% dry weight (DW) (Cai et al. 2013), in which the lipid can be converted into biodiesel (Chisti 2007; Lam and Lee 2011; Singh et al. 2017). According to Tsukahara and Sawayama (2005), a lipid content of 30% in microalgae cells is equivalent to an annual lipid production of 4.5–7.5 ton/ha. (4) They are a perfect candidate for the sequestration of CO_2_ and the reduction of GHGs. (5) They can utilize nutrients from most wastewaters, providing an alternative method for their treatment (Mureed et al. 2018; Li et al. 2019). (6) Residues of microalgal biomass, after lipid extraction, can be utilized as a source of nitrogen, as a protein-rich food, or crop fertilizer (Li et al. 2019).

Chlorophyta species are a good candidate for the remediation of effluent coming from urban and rural areas (Katiyar et al. 2021; Kumari et al. 2021). Among these, *Chlorella fusca* is a species with high productivity in cultures at different scales (Jerez et al. 2014, 2016). However, it is important to continue studying the possible use of *C. fusca* biomass, to depurate UW, mitigate eutrophication in aquatic ecosystems, and decrease economic costs of both the treatment of UW and biomass production (Peralta et al. 2019).

Based on this background, in the present study we analyzed the biomass production of *C. fusca* exposed to UW, as well as contaminant removal efficiency, the lipid and protein content, and the electron transport rate and maximum quantum yield, both considered indicators of microalgal physiological status. In vivo chlorophyll a fluorescence has been successful used as estimator of photosynthetic capacity in *C. fusca* in different type of photobioreactors and it has been related to biomass productivity and accumulation of bioactive compounds (Jetrez et al., 2016; Peralta et al., 2019).The use of effluents as culture media to produce metabolites of interest has the multiple purpose of contributing to their purification, through the degradation of organic and inorganic contaminants, and valuing them to obtain a product of interest.

## Materials and methods

### Microalgae strain

*Chlorella fusca* (Shihira and Krauss 1965) (Chlorophyta) (UTEX 343) was obtained from the Culture Collection of Algae at the University of Texas (Austin, USA) and is maintained at the Photobiology laboratory, University of Malaga.

### Urban wastewater collection

UW was collected from the Guadalhorce Wastewater Treatment Plant (WWTP) in Málaga city, located between 36° 40′ 56.24’’N and 4° 28′ 22.19’’W (Málaga, Spain) and stored at 4 °C in the dark until needed for the experiments. At this WWTP, suspended solids, non-biodegradable wastes and chemical contaminants are removed through subsequent physical, chemical, and biological processes. The generated sludge is treated and converted into compost, while the liquid effluent resulting from centrifugation must be reintroduced into the system to continue the treatment process. These liquid effluents from UW treatment were used in the present study.

### Experimental design and culture conditions

Fifteen polyvinyl methacrylate (Plexiglass XT-29080, non-filtering photosynthetically active radiation (PAR) and UV radiation) 1.5 l cylindrical reactors were prepared and divided into five treatments (with three replicates each). One of them was the control containing 1 l of culture medium (100% CM) (Sorokin and Krauss 1958), prepared with distilled water and the following composition (g l^−1^): KNO_3_, 1.25; KH_2_PO_4_, 1.25; MgSO_4_ 7H_2_0, 1.00; CaCl_2_, 0.0835; H_3_BO_3_, 0.1142; FeSO_4_ 7H_2_0, 0.0498; ZnSO_4_ 7H_2_0, 0.0882; MnCl_2_ 4H_2_0, 0.0144; MoO_3_, 0.0071; CuSO_4_ 5H_2_0, 0.0157; Co(NO_3_)_2_ 6H_2_0, 0.0049; ethylenediaminetetraacetic acid (EDTA), 0.5. The pH of the medium was 6.8. The other four treatments contained different percentages of CM (Sorokin and Krauss 1958), with all the macro- and micronutrients but without nitrogen compounds (KNO_3_, 1.25 g l^−1^), to promote nitrogen use from the wastewater (Markou and Nerantzis 2013), to which 25%, 50%, 75%, and 100% of UW was added, i.e., 25% UW + 75% CM, 50% UW + 50% CM, 75% UW + 25% CM, and 100% UW. The UW (100%) contained nitrogen, mainly in the form of ammonium (NH_4_^+^) (620 mg l^−1^), Cl^−^ (455 mg l^−1^), PO_4_^3−^ (119.8 mg l^−1^), SO_4_^2−^ (2.7 mg l^−1^), Na^+^ (271 mg l^−1^), K^+^ (104.6 mg l^−1^), Mg^2+^ (89.3 mg l^−1^) and Ca^2+^ (149 mg l^−1^). UW chemical composition was analyzed by ion chromatography (883 Basic IC plus, Metrohm, Switzerland).

The initial cultures contained 5 × 10^6^ cells ml^−1^ of *C. fusca*. Bioreactors were maintained at uniform and continuous aeration (air flow, 0.15 l min^−1^) and were placed in a growth chamber with controlled photoperiod (12L:12D), irradiance (150 µmol photons m^−2^ s^−1^) and temperature (30 °C). On day 0 and 14 the biological oxygen demand (BOD_5_) was analyzed and, on the freeze-dried samples of microalgae, the carbon and nitrogen contents, and the protein and lipid content were determined. On days 0, 3, 7, 10, 12, and 14, 45 ml of culture was removed for pH measurement and cell count. On days 0, 10, and 14 the physiological status of cultures and photosynthetic pigment content, and on days 0, 3, 10, and 14 dissolved organic carbon (DOC) and total nitrogen (TN) were determined.

### Estimation of growth parameters and culture conditions

Growth was monitored throughout the whole experiment by cell counting, i.e., three 50 μl replicates were sampled at initial time (0 h), at 3, 7, 10, 12, and 14 days, and the cells were counted using Neubauer chamber according to Utermöhl (1958). The pH was determined with a Crison pH meter at each time tested. Algal density was expressed as cell ml^−1^. With these results, a growth curve was constructed for each of the trials. Finally, the intrinsic growth rate and the doubling time were calculated, using the following equations (Reynolds 1984):

$$\mu = {{\ln \left( {{{N_{2} } \mathord{\left/ {\vphantom {{N_{2} } {N_{1} }}} \right. \kern-\nulldelimiterspace} {N_{1} }}} \right)} \mathord{\left/ {\vphantom {{\ln \left( {{{N_{2} } \mathord{\left/ {\vphantom {{N_{2} } {N_{1} }}} \right. \kern-\nulldelimiterspace} {N_{1} }}} \right)} {t_{2} - t_{1} }}} \right. \kern-\nulldelimiterspace} {t_{2} - t_{1} }}$$

where N_2_ and N_1_ are the final and initial cell density (cells ml^−1^) at final (t_2_) and initial (t_1_) time interval (days).

$$Doubling\,time:\,\,DT = \ln {{\left( 2 \right)} \mathord{\left/ {\vphantom {{\left( 2 \right)} \mu }} \right. \kern-\nulldelimiterspace} \mu }$$

### Carbon and nitrogen content in the biomass of *C. fusca*

Carbon and nitrogen content analyses were carried out on the freeze-dried samples of microalgae from each of the bioreactors, extracted on the day 14th of the experiment, as well as on the samples of the starting inoculum from the original culture. Samples were processed based on a CHN (carbon, hydrogen, nitrogen) analysis. Total C and total N were determined from dry biomass, using a CHN Perkin-Elmer 2400 elemental analyzer, with He as carrier gas, in which C was oxidized at 600 °C. The goodness and precision of the analyses performed were determined using certified EDTA (n = 10, the standard deviation was 0.04 and 0.08 for C and N respectively).

### Protein and lipid content in the biomass of *C. fusca*

The nitrogen content was converted into protein concentration values using the conversion factor for microalgae (6.25) (Lourenço et al. 2002). Lipid content of the lyophilized biomass was determined according to the extraction method based on the use of a mixture of chloroform and ethanol (2:1 by volume) with 0.01% BHT (Folch et al. 1957).

### *Organic and inorganic compound removal analysis of urban wastewater: DOC, TN, and BOD*_*5*_

For DOC and TN analyses, the supernatant from each incubation was collected and filtered (precombusted GF/F filter). DOC and TN were measured in a Shimadzu TOC-L Analyzer, using the non-purgeable organic carbon (NPOC) method and TN, respectively. Samples were purged for 2.5 min at 150 ml min^−1^ flux with zero synthetic air (79% N and 21% O_2_) and acidified with 1.5% HCl. The determinations were performed in complete combustion of the samples at 720 °C in an oven with a Pt catalyst and by subsequent detection of the resulting gases in an IR detector (DOC) and fluorescence (TN). A five-point calibration curve with potassium-hydrogen-phthalate (KHP) and potassium nitrate (Sigma), ranging between 5 and 100 mg l^−1^, was used for DOC, and between 1 and 20 mg l^−1^ for TN. Two distilled water blanks were run between samples to ensure that there was negligible carryover between samples, and five technical replicates of each sample were analyzed, and three real daily replicates were made. The average coefficient of variation for fivefold DOC analysis was 1–2% of the mean. The standards used for the measurements were USGS 40, USGS 41, IAEA N1. The percentages of recovery were USGS 40: 100.6% (for carbon value) and 61.20% (for nitrogen), USGS 41: 123% (for carbon value) and 82.76% (for nitrogen). Three replicates of each treatment were analyzed, i.e., one sample per bioreactor and day. BOD_5_ was analyzed at the beginning (day 0) and end (day 14) of the experiment using an OxiTop-Control developed by WTW (Wissenschaftlich-technische Werkstätten GmbH, Weilheim, Germany).

The nutrient removal percentage was calculated according to the following equation:

$$\% \,Nutrient\,removal\,efficiency\, = \,\left[ {{{\left( {C_0 - C_F} \right)} \mathord{\left/ {\vphantom {{\left( {C_0 - C_F} \right)} {C_0}}} \right. \kern-\nulldelimiterspace} {C_0}}} \right]\,\, \times \,100$$

where C_0_ and C_F_ stand for the initial concentration at the beginning of the experiment (day 0) and final concentration at the end of the experiment (day 14), respectively.

### Pigment concentration

The concentration of photosynthetic pigments (chlorophyll *a* (Chl *a*), chlorophyll *b* (Chl *b*), and carotenoids) was calculated spectrophotometrically after adding 2 ml of dimethylformamide (DMF) to 1 mg of the lyophilized sample. The sample was kept overnight in the dark at 4 °C, then it was centrifuged and analyzed at different wavelengths (750, 664, 647, and 480 nm, UV–Vis spectrophotometer Shimadzu UV-16-03). The Chl *a* and Chl *b*, as well as total carotenoids, were calculated according to Wellburn (1994). The results were expressed as µg ml^−1^; the ratio of Chl *a*/Chl *b* and pigment index (ratio of carotenoids/Chl *a*) were calculated (Bodnar et al. 2016, 2019).

### Photosynthetic activity

The physiological status of cultures was evaluated by analyzing the photosynthetic capacity by fluorescence of Chl *a* associated with photosystem II (PSII) in 10 ml culture samples after 15 min in the dark, using a noninvasive pulse amplitude modulation fluorometer PAM-2000 (H. Walz, Germany) supplied with red light-emitting diodes (maximum wavelength at 630 nm) in a light-tight and mixed measuring chamber placed on a magnetic stirrer. The minimum fluorescence level (Fo) was determined using a weak modulated light (< 0.15 μmol photons m^−2^ s^−1^, 0.5–1 kHz). A single high intensity flash (> 10,000 μmol photons m^−2^ s^−1^, 0.6 s duration) was used to achieve the maximum fluorescence level both in the dark (Fm) and light (Fm’) adapted state. Maximal quantum yield (Fv/Fm) was determined as Fv/Fm = (Fm − Fo)/Fm in the dark-adapted samples. The relative electron transport rate (rETR, μmol electrons m^−2^ s^−1^) was calculated as x Y(II)* E_PAR_, where Y(II) is the effective quantum yield of PSII calculated as Fm’-F/Fm’ being Fm’ the maximal fluorescence of algae under light conditions and the Ft the and *Ft*, the steady state chlorophyll fluorescence under actinic light. E_PAR_ is the incident PAR irradiance. YII was determined under different intensities of actinic light (Figueroa et al. 2013).

### Statistical treatment

One-way and two-way repeated measures ANOVA were performed to distinguish significantly different results between treatments with different UW concentrations and exposure times. All data represent the mean (± standard deviation, SD) of the treatments that were performed in triplicate. Post hoc comparisons were tested when significant differences were found (95%, α = 0.05). All statistical assessments were carried out using the Statistica software (version 10, Statsoft Poland).

## Results

### Analysis of *C. fusca* growth in urban wastewater

At the end of the assay, a greater cell increase was observed in the culture subjected to 50% UW concentration (65.5 × 10^6^ cell ml^−1^), followed by the treatments 100% UW (23.6 × 10^6^ cell ml^−1^), 75% UW (15.6 × 10^6^ cell ml^−1^), the control (100% CM) (11.8 × 10^6^ cell ml^−1^), and finally 25% UW (1.4 × 10^6^ cell ml^−1^).

**Fig. 1:**
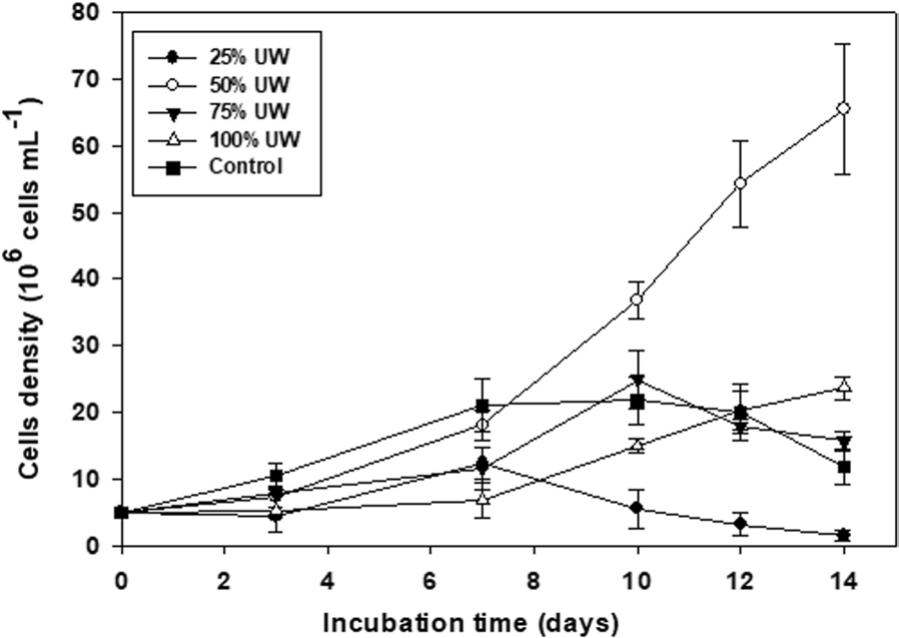
Growth evolution of *C. fusca* (cells ml^−1^) in the different treatments (control, 25% UW, 50% UW, 75% UW, and 100% UW), throughout the incubation period (14 days). Data are expressed as mean ± standard deviation (n = 3)

The density recorded in the control exceeded that of the UW treatments until day 7, remaining similar on days 10 and 12, decreasing at the end of the experiment possibly due to nutrient depletion. The cultures with 25% UW reached their highest growth (in terms of cell density) on day 7, followed by a decrease in the number of cells, possibly due to the depletion of nutrients in the medium and contamination by different species of protozoa. In the treatment with 50% UW, a successive increase in growth was recorded until the end of the experiment. In the treatments with 75% UW, an increasing density was observed until day 10 (although lower than that recorded at 50% UW), then a decrease was observed. In the treatment with 100% UW, the lowest density values ​​were recorded until day 10 (except for the treatment with 25% contaminated UW). On day 12, a cell density of 20.3 × 10^6^ cell ml^−1^ was recorded (similar to the control and treatment with 75% UW), increasing slightly on the last day of the experiment (23.6 × 10^6^ cell ml^−1^). A lag phase during the first 3 days was observed in the 100% UW and 25% UW treatments (Fig. 1). The doubling time was 4.3 days, 3.7 days, 4.5 days, and 6.2 days, for control, 50% UW, 75% UW, and 100% UW, respectively.

**Fig. 2:**
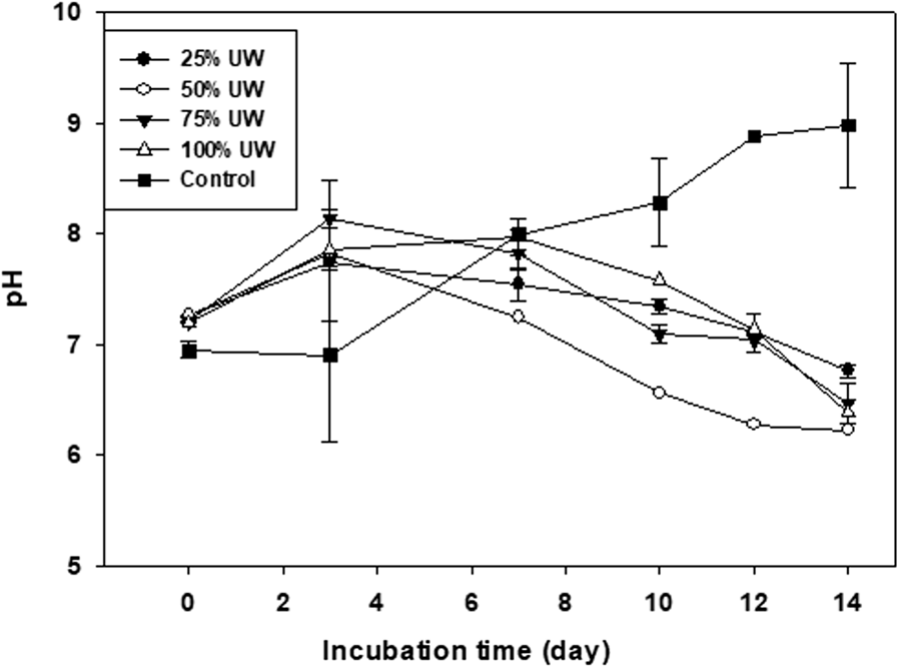
Evolution of pH throughout the incubation period (14 days), in the different treatments (control, 25% UW, 50% UW, 75% UW, and 100% UW). Data are expressed as mean ± standard deviation (n = 3)

Figure 2 shows the pH variations recorded during the period studied. The pH mean values (min. and max.) were: 6.9 – 9.3 (control); 6.7 – 7.7 (25% UW); 6.8 – 8 (50% UW); 6.4 – 8.1 (75% UW), and 6.7 – 7.9 (100% UW).

ANOVA test showed a significant contrast at one level for the three interactions (number of cells recorded in the counts, time, and UW concentrations). The post hoc analysis revealed a marked difference in means for the treatment subjected to 50% UW.

### Protein concentration and C/N ratio in the biomass of *C. fusca*

**Fig. 3:**
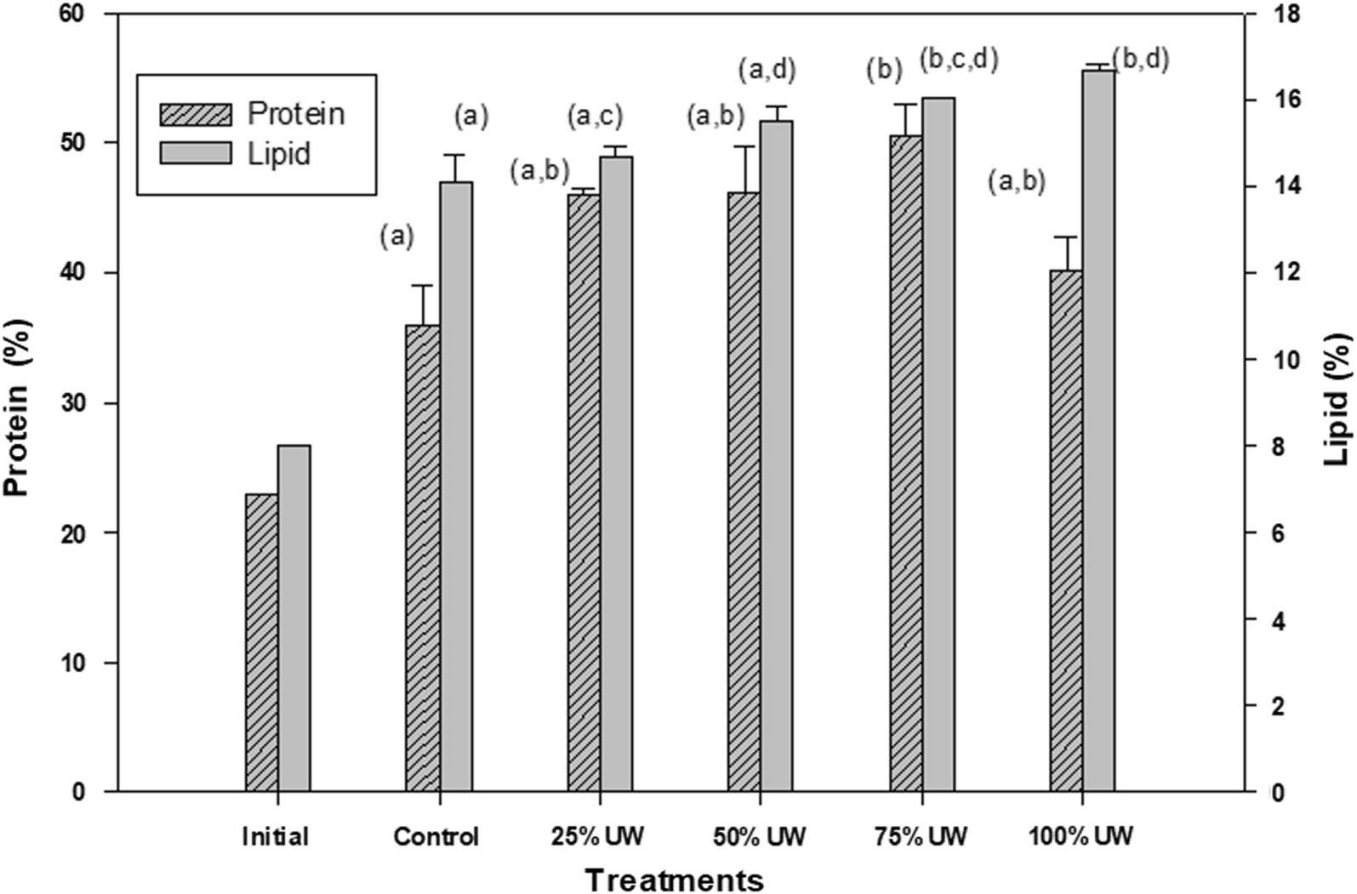
Protein (grated bars) and lipid (solid bars) percentage contained in *C. fusca* in the different treatments (control, 25% UW, 50% UW, 75% UW, and 100% UW) at the end of the incubation period (14 days). The same letters indicate no significant differences between the groups (*p* > 0.05). Data are expressed as mean ± standard deviation (n = 3)

The initial protein concentration was 22.9%. A progressive increase in the protein concentration with increasing UW concentration was observed in the cultures, reaching a maximum value in the 75% UW treatment, with a 50.5% increase of production. In 100% UW treatment, the protein percentage was lower than in the other treatments (40%), and very similar to the control (Fig. 3).

The carbon and nitrogen concentrations allowed performing an analysis of the C/N ratio in the cultures studied. It is noteworthy that the C/N ratio was very low, indicating a high proportion of N in microalgae samples. The C/N ratio (average ± SD) obtained in the harvested biomass at the end of the incubation period in the inoculums and different treatments was: 6.06 (± 0.02), 6.53 (± 0.09), 6.9 (± 0.08), 6.07 (± 0.53), 5.93 (± 0.002), and 6.2 (± 0.16), for inoculum, the control, 25% UW, 50% UW, 75% UW, and 100% UW treatments, respectively. No statistically significant differences were observed between controls and treatments (p > 0.05).

### Lipid content in the biomass of *C. fusca*

The initial lipid percentage was 8%. At the end of the incubation period, the percentage of intracellular lipids increased significantly compared to the control (*p* < 0.05) when UW was added (14.7% in 25% UW treatment, 15.5% in 50% UW, 16% in 75% UW, and 16.7% in 100% UW) (Fig. 3).

### *Removal efficiency: DOC, TN, and BOD*_*5*_

**Fig. 4:**
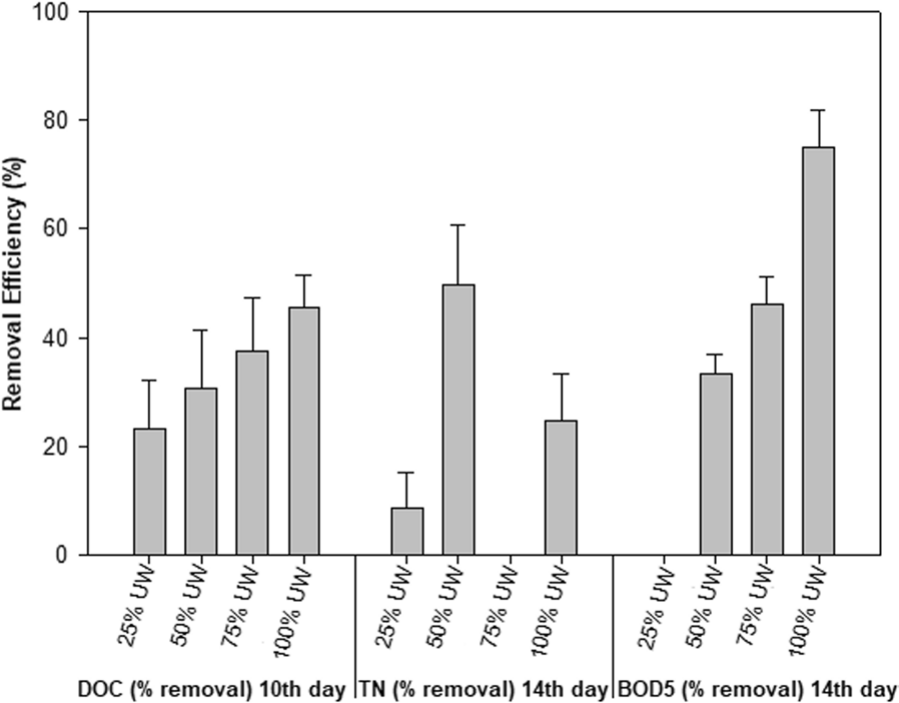
Maximum removal efficiency (%) of DOC and TN on the 10th day and BOD_5_ on the 14th day in the different treatments (25% UW, 50% UW, 75% UW, and 100% UW). Data are expressed as mean ± standard deviation (n = 3)

The DOC and TN removal efficiency was calculated on the third day of incubation, on the tenth day, and at the end of the experiments (14th day). The BOD_5_ removal efficiency was calculated only at the end of the experiments (14th day). Figure 4 shows the percentage removal of nutrients from UW at times when efficiency was the highest (10th day for DOC and 14th day for TN and BOD_5_).

The initial DOC concentrations ranged between 12.08 mg L^−1^ (± 0.25) and 14.64 mg L^−1^ (± 0.68), TN between 2.73 mg L^−1^ (± 0.08) and 7.31 mg L^−1^ (± 0.08), and BOD_5_ between 250 mg L^−1^ (± 0) and 970 mg L^−1^ (± 70.7). On the tenth day, DOC values decreased in all treatments, obtaining the maximum remediation efficiency (23.35%, 30.63%, 37.37%, and 45.48%, for 25% UW, 50% UW, 75% UW, and 100% UW, respectively).

In the control, where the nitrate source came from the CM, the TN concentration diminished on the seventh day of incubation. In the rest of the treatments, the source of nitrogen was fundamentally ammonium. When the algae began to degrade these sources of ammonium into more assimilable forms, the nitrogen of the medium increased markedly in the treatments with the highest UW concentrations. Once the ammonium was exhausted, the microalgae would consume the nitrate. The treatment with less UW (25% UW) maintained its level of TN on the third day to increase on the tenth and decrease on day 14 with respect to the initial value. However, in the treatments with the highest amount of UW, TN increased until the tenth day to significantly decrease on day 14 (removal efficiency: 55% and 24.6%, for 50% UW and 100% UW, respectively) (Fig. 4).

ANOVA test showed that the results for both variables (DOC and TN) were statistically significant (*p* < 0.05), being affected by time and UW concentration factors. The post hoc analysis of DOC results showed a significant difference in the behavior of the 25% UW treatment compared to the rest. In the case of TN, the effects on the mean were markedly different among all treatments (*p* < 0.05).

BOD_5_ values decreased, except in the 25% UW treatment. At the end of the incubation time, in the treatment with 25% UW, the number of *C. fusca* cells decreased and, simultaneously, contamination by different species of protozoa, probably coming from the UW itself, was observed. The increase in the final BOD_5_ value only confirmed the increasing contamination. In the rest of the treatments, BOD_5_ decreased more sharply as the amount of UW increased (33%, 46%, and 75% in 50% UW, 75% UW, and 100% UW treatments, respectively) (Fig. 4).

### Physiological status of *C. fusca*

#### Photosynthetic pigments

Chl *a* level increased markedly in the control and 75% UW with the exposure time. In 25% UW treatment, the maximum Chl *a* concentration was reached on the tenth day of incubation, decreasing at the end of the analyzed period until values below the initial ones. In the other treatments (50% UW and 100% UW), Chl *a* concentration increased until the tenth day of incubation and decreased at the end of the incubation period but was finally bigger than the initial values (Table 1).

**Table 1:** Chlorophyll *a* (Chl *a*), Chlorophyll *b* (Chl *b*) and Carotenoids (Carot) (µg ml^−1^) evolution along the incubation time (0, 10 and 14 days) in the different treatments (Control, 25% UW, 50% UW, 75% UW and 100% UW)

	Control			25% UW			50% UW			75% UW			100% UW		
Days	Chl *a* (µg ml^−1^)	Chl *b* (µg ml^−1^)	Carot (µg ml^−1^)	Chl *a* (µg ml^−1^)	Chl *b* (µg ml^−1^)	Carot (µg ml^−1^)	Chl *a* (µg ml^−1^)	Chl *b* (µg ml^−1^)	Carot (µg ml^−1^)	Chl *a* (µg ml^−1^)	Chl *b* (µg ml^−1^)	Carot (µg ml^−1^)	Chl *a* (µg ml^−1^)	Chl *b* (µg ml^−1^)	Carot (µg ml^−1^)
0	0.62 ^(a)^ (± 0.17)	0.31^(a)^ (± 0.09)	0.08 ^(a)^ (± 0.01)	0.70 ^(ad)^ (± 0.11)	0.36 ^(a)^ (± 0.15)	0.13 ^(a)^ (± 0.02)	0.57 ^(a)^ (± 0.16)	0.36 ^(a)^ (± 0.03)	0.13 ^(a)^ (± 0.02)	0.53 ^(a)^ (± 0.09)	0.33 ^(a)^ (± 0.15)	0.13 ^(a)^ (± 0.02)	0. 49 ^(a)^ (± 0.10)	0.32 ^(a)^ (± 0.18)	0.13 ^(a)^ (± 0.02)
10	10.13 ^(b)^ (± 0.08)	3.96 ^(b)^ (± 0.17)	2.24 ^(b)^ (± 0.21)	4.22 ^(e)^ (± 0.56)	1.41^(c)^ (± 0.10)	0.94 ^(c)^ (± 0.27)	10.34 ^(bc)^ (± 0.08)	3.38 ^(b)^ (± 0.61)	1.34 ^(b,c)^ (± 0.96)	8.40 ^(c)^ (± 0.97)	3.15 ^(b)^ (± 0.29)	1.89 ^(b,c)^ (± 0.32)	3.45 ^(e)^ (± 0.41)	1.54 ^(c)^ (± 0.03)	0.70 ^(c)^ (± 0.06)
14	11.34 ^(b)^ (± 0.08)	3.80 ^(b,d)^ (± 0.57)	1.32 ^(b)^ (± 0.94)	0.46 ^(d)^ (± 0.24)	0.20 ^(a)^ (± 0.15)	0.11 ^(a)^ (± 0.06)	7.44 ^(c)^ (± 0.56)	4.17 ^(d)^ (± 0.48)	1.90 ^(b,c)^ (± 0.87)	11.38 ^(b)^ ± 1.08)	4.17 ^(d)^ (± 0.31)	2.40 ^(b)^ (± 0.40)	2.87 ^(e)^ (± 0.36)	2.06 ^(c)^ (± 0.10)	0.58 ^(c)^ (± 0.01)

Data are expressed as mean ± standard deviation (n = 3)

^(a, b, c, d, e)^ The same letters indicate no significant difference between the groups (*p* > 0.05).

Chl *b* levels increased markedly in the control and 25% UW treatment until the tenth day of incubation. In the control, there was no difference between Chl *b* concentration on the 10th and 14th days. In 25% UW treatment, Chl *b* decreased at the end of the incubation period. In the other treatments, Chl *b* levels increased markedly with the exposure time. Carotenoid concentration in the control and in the 25% UW and 100% UW treatments during the incubation period followed the same pattern as Chl *b*. In 50% UW and 75% UW treatments, carotenoid concentration increased until the end of the incubation period (Table 1).

UW addition to the culture medium modified the Chl *a*/Chl *b* and carotenoid/Chl *a* ratio. Although the Chl *a* content was higher than that of Chl *b* in all the samples, the Chl *a*/Chl *b* ratio decreased with rising UW concentration and with the exposure time. Inversely, in the control and 75% UW treatment it increased over time. The pigment index decreased in the control, remained similar over time in the treatments with 100% UW, and increased in the treatments with 25% UW, 50% UW and 75% UW (Table 2).

**Table 2:** Pigments index and chlorophylls *a*/*b* ratio evolution along the incubation time (0, 10 and 14 days) in the different treatments (Control, 25% UW, 50% UW, 75%UW and 100%UW)

Ratio Ch *a*/Ch *b*					
Days	Control	25% UW	50% UW	75% UW	100% UW
0	2	1.94	1.58	1.61	1.53
10	2.56	2.99	3.06	2.67	2.24
14	2.98	2.3	1.78	2.73	1.39
Pigment index (Carotenoids/Chl *a*)					
Days	Control	25% UW	50% UW	75% UW	100% UW
0	0.13	0.19	0.23	0.25	0.27
10	0.22	0.22	0.13	0.23	0.2
14	0.12	0.24	0.26	0.7	0.2

#### In vivo chlorohyll a fluoprescence with photosystem II

**Fig. 5:**
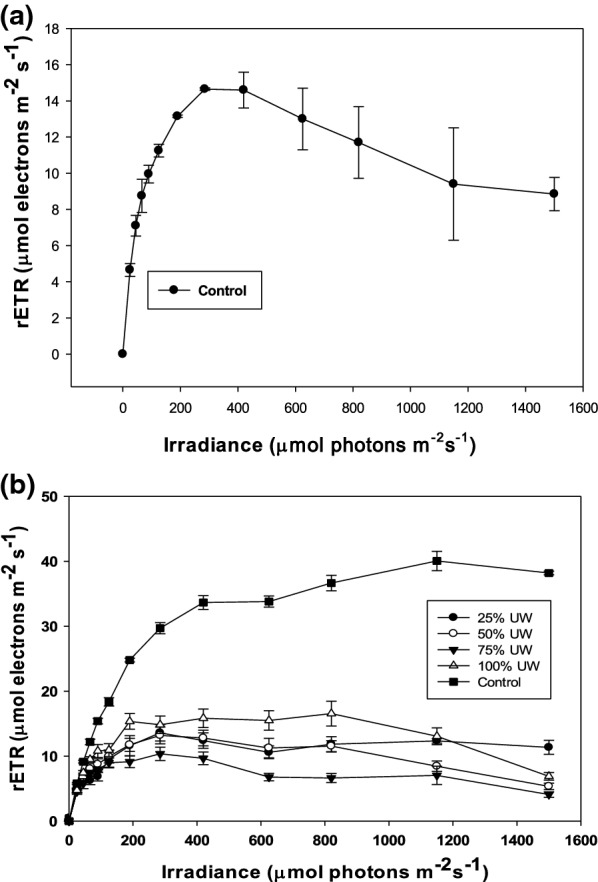
Initial values in the control—day 0—(**a**) and final values—day 14—in the control and UW treatments (**b**) of the electron transport rate (rETR) as a function of irradiance. Data are expressed as mean ± standard deviation (n = 3)

The photosynthetic pattern, based on the various parameters obtained by measuring the fluorescence, was evaluated. The ETRmax value of all treatments followed an increasing pattern from the time of inoculation (its lowest point for all cultures) until final processing. Figure 5 shows the initial values of the control (a) and final—day 14—in the control and UW treatments (b) of electron transport rate (rETR) as a function of irradiance for each treatment. It was observed that the photosynthetic efficiency (initial slope of the function rETR-irradiance) was lower under UW than that in the control (Fig. 5). rETR decreased under all UW treatments and a decrease of rETR with the irradiance increasing in 100%UW was observed indicating the existence of photoinhibition.

**Fig. 6:**
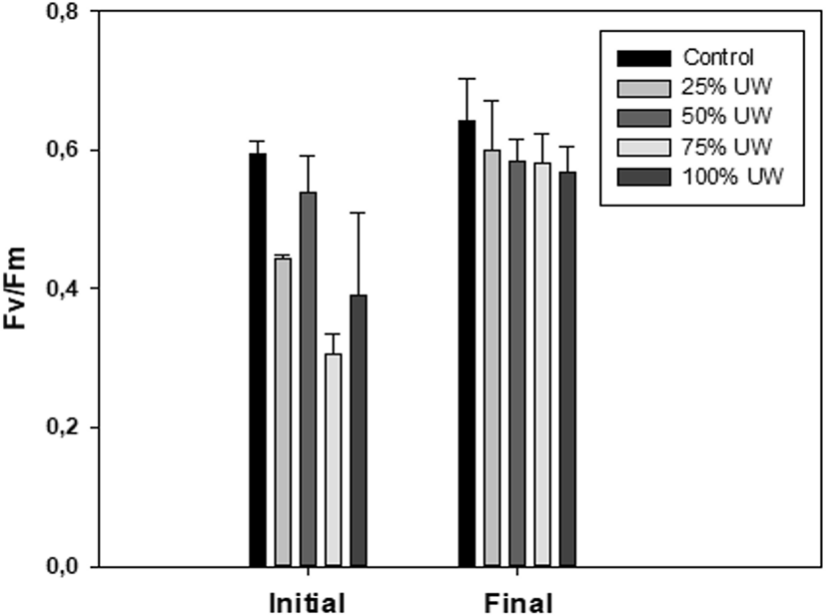
Initial and final maximum quantum yield (Fv/Fm) for each treatment. Data are expressed as mean ± standard deviation (n = 3)

On the first day, the cultures that showed higher Fv/Fm values were those with lower UW concentration, i.e., 25% UW (0.44 ± 0.005) and 50% UW (0.54 ± 0.05). On the day of inoculation, the Fv/Fm value in 75% UW (0.31 ± 0.03) was lower than in 100% UW (0.39 ± 0.1), but from day 10 it was observed that the Fv/Fm value for 75% UW exceeded that of 100% UW, forming a scale of decreasing values, as a function of a higher concentration of UW. Again, as with ETRmax, on day 14 the Fv/Fm values in the treatments with UW were higher than on day 10: the control, 0.64 ± 0.06; 25% UW, 0.6 ± 0.07; 50% UW, 0.58 ± 0.03; 75% UW, 0.58 ± 0.04, and 100% UW, 0.57 ± 0.03 (Fig. 6). ANOVA test showed significant differences between the mean values obtained for Fv/Fm (*p* < 0.05), which implies that both the UW concentration and time influence Fv/Fm values.

## Discussion

Microalgae have lately attracted great interest worldwide due to their application potential in wastewater remediation, in nutraceutical, pharmaceutical, and renewable energy industries (Pahazri et al. 2016; Khan et al. 2018). Different researchers demonstrated the purification capacity of microalgae and verified that the biomass obtained had a high content of bioproducts (Tang et al. 2020).

On the other hand, the use of wastewater as a substitute for algae nutrients would significantly reduce the operational cost of the cultures. Wastewater contains phosphorus, nitrogen, carbon, and other constituents needed for microalgal growth, although some other undesirable compounds such as emerging pollutants and heavy metals can also be found (Morales-Amaral et al. 2015).

In this sense, Khan et al. (2018) described the advantages of microalgae to produce biofuels and various bioactive compounds and discussed culturing parameters. According to these authors, the most important and challenging issues are increasing microalgal growth rate and enhancing bioproduct synthesis, among others.

In the present study, we investigated the ability of *C. fusca* to grow in a medium composed of UW, as well as its efficiency for the removal of contaminants and its potential to produce biomass with a high lipid and protein content.

The results show that the final cell growth values in the 50% UW, 75% UW, and 100% UW treatments were higher than in the control. In cultures with 50% UW, the highest cell density and the shortest doubling time were obtained (Fig. 1). According to Katiyar et al. (2021) *Chlorella minutissima* and *Chlorella sorokiniana* showed higher growth rate, lipid content, and biomass productivity, when cultured in wastewater than in control. Similar results were reported by Singh et al. (2017), although these authors recorded a higher growth rate and biomass production, and shorter doubling time, using *Parachlorella kessleri*-I in 100% municipal wastewater concentration.

This greater growth in the treatments with UW is possibly because the concentration of ammonium as a nitrogen source prevents this nutrient from being limiting (Gómez Serrano 2012). Lin et al. (2007) observed that *Chlorella pyrenoidosa* (LK) was ammoniacal-N tolerant, with cell density increases in leachate with 405 mg l^−1^ of NH_4_^+^. Similar results were observed in this study, where the cell density of *C. fusca* increased in UW with about 620 mg l^−1^ of ammonium. In turn, the UWs provided a greater amount of nutrients to the treatments, including PO_4_^3−^, SO_4_^2−^, K^+^, Mg^2+^ (among others) favoring growth under these conditions. However, the nutrients are not the sole requirement for microalgal development, since temperature, light, aeration, and pH, as well as mixing, can contribute to their growth (Pahazri et al. 2016).

In algal cultures, the pH usually increases due to photosynthetic CO_2_ assimilation. This pH increase can be compensated by respiration (Jyoti and Awasthi 2013). According to this, in our study, pH values would be expected to increase along the experiment due to photosynthetic activity; however, the pH increased only in the control, while in cultures with UW, it went down, despite their photosynthetic activity (Fig. 2).

Nitrogen is usually present in wastewater as NH_4_^+^, contributing significantly to the changes in pH value. Assimilation of nitrate ions by algae tends to raise the pH, but if ammonia is used as nitrogen source, the pH of the medium may decrease (Kong et al. 2011; Pahazri et al. 2016). Scherholz and Curtis (2013) analyzed the influence of ammonium and pH on the growth of *Chlorella vulgaris* in photobioreactors and observed that cultures provided with 4.5% nitrogen from ammonium showed significant growth. At the same time, they observed a decrease in pH during the growth phase, followed by a pH rise indicating sequential ammonium and nitrate metabolism. These results are consistent with the almost exclusive consumption of ammonium instead of nitrate and corroborate the statements of previous works where it is mentioned that the use of nitrate is inhibited in the presence of ammonium (Florencio and Vega 1983).

In the 1970s, the worldwide energy crisis encouraged the use of microalgae as renewable and sustainable sources to produce biofuels. Depending on the type of wastewater used in the cultivation of microalgae, the lipid percentage obtained will be different. In agricultural wastewaters *Chlorella* sp. showed 9% DW (Jacobson and Alexander 1981) and 13.6% DW (Wang et al. 2010a) of lipid content. In industrial effluents, *Chlorella saccharophila* was obtained with 18.10% DW of lipid content (Chinnasamy et al. 2010). In this study, the lipid percentage was 14.7% DW (25% UW), 15.5 DW (50% UW), 16% DW (75% UW), and 16.7% DW (100% UW). These values are comparable to those obtained by Jebali et al. (2015) and Hernández et al. (2016). Hempel et al. (2012) showed that the strains with the highest lipid content were *Chlorella* sp. 589 (30.2% DW), *C. saccharophila* 477 (27.6% DW), and *Chlorella* sp. 800 (24.4% DW).

It is known that the production of lipids increases up to 65% by the nutritional deprivation of microalgae (Markou and Nerantzis 2013). In our case, we observed a smaller increase. This is because, despite being the original purpose, the high concentration of ammonium in the UW and the ability of *C. fusca* to metabolize it showed it was not in a status of nutritional nitrogen deprivation. The increase in lipid production is possibly related to the stress conditions induced by physicochemical conditions of the UW. Probably the solution may be to perform assays in two phases: the first to decrease the concentration of nutrients, essentially nitrogen, and a second crop in which, with nitrogen deficiency, lipid production increases. This solution was also given by Cai et al. (2013).

Moreover, it has been recorded that the maximum accumulation of lipids in *Chlorella* sp. is related to the pH of the medium, the optimum being pH values ​​between 7.0 and 8.5 (Wang et al. 2010b; Sakarika and Kornaros 2016). In our study, the pH range in the treatments with UW was 6.4 – 8.1, therefore this parameter should be considered in later studies, in order to increase the productivity of lipids in *C. fusca*.

The cultivation of algae has expanded to new fields, such as feed and food, cosmetics, and biopharmaceutical products (Khan et al. 2018). In this regard, different authors investigated biomass productivity and the synthesis of amino acids on microalgae strains. Hempel et al. (2012) identified strains with an amino acid content of more than 40% DW: *Spirulina platensis* reached a protein content of 46.8% DW, *Chlorella* sp. 589 of 44.3% DW, and *C. saccharophila* 477 of 42.4% DW. In our study, a higher concentration of UW in the cultures leads to an increase in the production of proteins, implying a greater assimilation of nitrogen, which in the UW is mainly ammonium (Peralta López 2013). Given the high proportion of protein accumulated in 75% UW treatment (51%), this system can be considered as a form of protein production for commercial use.

In addition to demonstrating that the biomass of microalgae obtained in cultures with wastewater has a high content of bioproducts, these authors verified their capacity and efficiency for bioremediation. Although *C. fusca* is not a species commonly used in the remediation of UW (Pahazri et al. 2016), we demonstrate that it has the capacity to grow mixotrophically and accumulate nutrients from it. In this study, the maximum efficiency of DOC removal was obtained on the tenth day of exposure, with removal efficiency ranges that vary between 23.35% and 45.48% (Fig. 4). Katiyar et al. (2021) reported a higher total organic Carbon removal efficiency by *C. minutissima* and *C. sorokiniana* (95% and 98%, respectively) in wastewater collected from India (500 ml, for 12 days), compared to that recorded in this study.

This trend is justified by different authors: Eny (1951) observed that the metabolic route of *Chlorella* sp. could be altered with the supply of organic substrates such as glucose or organic acids, which means that they can perform not only autotrophic but also heterotrophic growth. The organic compounds can be used as an essential nutrient (Sachdev and Clesceri 1978) or as an accessory growth factor (Saunders 1957). The heterotrophic growth of microalgae (including *Chlorella* sp.) can be rapid, from the incorporation of organic substrates in the oxidative assimilation process for storage material production (Burrell et al. 1984).

In this study, TN decreased at the end of the incubation period (14th day) in the 50% UW and 100% UW treatments, obtaining a removal efficiency between 55% and 24.6%. Similar percentages were reported by Katiyar et al. (2021), with TN removal rates (12 days) for *C. minutissima* and *C. sorokiniana* as 28.46% and 40% respectively. An opposite tendency was observed by Lin et al. (2007), who reported that the relative NH_4_^+^ removal rate in lower leachate concentrations (10% and 30%) was higher than that in higher concentrations (50%, 80%. and 100%). In this sense, *C. fusca* is considered suitable microalgae for the degradation and elimination of nitrogenous waste present in UW.

The fact that microalgal cultures subjected to a high concentration of UW maintain their capacity for carbon removal is crucial for their possible use as a bioremediation system. The UW contains a microorganism cocktail (bacteria, protozoa, rotifers, among others) that feed on organic matter giving rise to a very high BOD_5_ (1000 mg l^−1^) (Madoni 2011). During the experiment, in the treatment with 100% UW, BOD_5_ decreased by 75% (from 1000 mg l^−1^ to 250 mg l^−1^). The dissolved organic matter in the UW is probably assimilated by microalgae and is thus eliminated. He et al. (2013) reported that the microalgae–bacteria consortium present in wastewater is more efficient in removing BOD (97%).

The dynamics of photosynthetic pigment content, changes in their ratio, as well as rETR and Fv/Fm associated with PSII, indicate *C. fusca* adaptation in response to the impact of UW addition to the culture medium. Different authors reported that the accumulation of Chl *a* is related to nitrogen metabolism (Rüdiger and López-Figueroa 1992). Chu et al. (2015) found that Chl *a* content in *C. vulgaris* cultures increased on the first days of incubation, when there was enough N in the medium, to decrease later when N was running low. In our case, the trend was similar, decreasing from day 10 until the end of the experiment. At the same time, in 50% UW and 75% UW concentrations, the carotenoid concentration increased at the end of the incubation period; similar results were reported by Kiran et al. (2014).

In this work, the increase in UW concentration was accompanied by a reduction of the Chl *a*/Chl *b* ratio and an increase in the pigment index (carotenoids/Chl *a*) in *C. fusca*. The Chl *a*/Chl *b* ratio can characterize the photochemical potential and biosynthetic activity of algae, controlling the absorbed light intensity (Tanaka and Melis 1997). Thus, under stress action, a decrease in Chl *a* content takes place, and accordingly the ratio between these two types of pigment decreases. When this happens, the pigment index increases due to the formation of carotenoids that perform a supporting and protective role in the photosynthesis. Chl *a* is the part of reaction centers and peripheral complexes of photosystems I and II, and Chl *b* is the component of the light-collecting complex of photosystem II. Therefore a change in the Chl *a*/Chl* b* ratio may indicate a shift of stoichiometric balance between the reaction center complexes of both photosystems and the light-collecting complex of photosystem II (Bodnar et al. 2016).

Also, the quality of the light inside each reactor was affected by the presence of UW, which possibly has a negative impact on cultures (Peralta et al. 2019). In this sense, the characteristics of the medium with different UW concentrations conditioned the biomass optical properties and consequently, the ETRmax and Fv/Fm. The UW treatments showed initial Fv/Fm values lower than the control, demonstrating the stress induced by the culture medium with UW at the beginning of the experiment, improving their yield at the end when manage to acclimate to the conditions of the culture medium. Similar results were obtained by Peralta et al. (2019), who reported that Fv/Fm was correlated with nitrogen content and maximal rETR with photosynthetic performance and nitrogen metabolism. The UW treatments reached the lowest rETR values at the end of the experiment in relation to the control. This indicates that microalgae could not achieve optimal photosynthetic activity, possibly due to the toxicity and turbidity of UW.

The cultivation of microalgae in UW is a promising alternative for its treatment, at the same time as it provides a culture medium with nutritional properties. Even though UW affected the photosynthetic activity of microalgae, they were able to grow and synthesize lipids and proteins. The TN, DOC, and BOD_5_ reduction efficiency increased with the exposure time, showing the potential of *C. fusca* to remove these compounds from effluents on a laboratory scale. Therefore, this is recommended as an eco-friendly method that should be tested for wastewater treatment on a larger scale, improving the factors limiting the performance of these microalgae-based wastewater treatment systems (Acién et al. 2016).

## Data Availability

The datasets generated during and/or analysed during the current study are available from the corresponding author on reasonable request.
